# Gamma gap and albumin-globulin ratio show poor sensitivity for monoclonal gammopathy screening in South Africa

**DOI:** 10.4102/ajlm.v14i1.2505

**Published:** 2025-03-26

**Authors:** Njabulo Khumalo, Cameron A. Francis, Siphiwe M. Baloyi, Jody A. Rusch

**Affiliations:** 1 Department of Chemical Pathology, National Health Laboratory Service, Cape Town, South Africa; 2 Division of Chemical Pathology, Faculty of Health Sciences, University of Cape Town, Cape Town, South Africa; 3 Department of Haematology, National Health Laboratory Service, Cape Town, South Africa; 4 Division of Clinical Haematology, Faculty of Health Sciences, University of Cape Town, Cape Town, South Africa

**Keywords:** gamma gap, globulins, paraproteins, paraproteinaemias, albumin/globulin ratio, inflammation

## Abstract

**Background:**

Monoclonal gammopathies, including multiple myeloma, present significant challenges in sub-Saharan Africa. Diagnosis is often missed because of limited screening tools. The gamma gap and albumin-globulin ratio (AGR) have been proposed as simple, cost-effective screening methods; however, their utility in settings with prevalent infectious and inflammatory diseases is unclear.

**Objective:**

This study evaluated the diagnostic accuracy of gamma gap and AGR in identifying patients who require further investigation for monoclonal gammopathies in South Africa.

**Methods:**

A retrospective analysis of 7946 patients who underwent investigations for monoclonal gammopathies at Groote Schuur Hospital, South Africa, between September 2015 and September 2022 was conducted. Patients were classified based on monoclonal protein detection, and the gamma gap, AGR, and multivariable models were evaluated for diagnostic performance.

**Results:**

Among the patients (median age: 61 years, 58% female [4632/7946] and 42% [3314/7946] male patients), 1231 had monoclonal proteins. A gamma gap cutoff of 46 g/L identified 35% of monoclonal cases (sensitivity), with 91% specificity and an area under the curve (AUC) of 0.60. The AGR showed a slightly better AUC of 0.63, with 44% sensitivity and 80% specificity at a 0.85 cutoff. Multivariable models incorporating age, sex, and hypogammaglobulinaemia improved performance, with the gamma gap model achieving an AUC of 0.73, improving the sensitivity to 58%, with a specificity of 78%.

**Conclusion:**

The gamma gap and AGR showed low sensitivity and moderate specificity in screening for monoclonal gammopathies, highlighting the need for integrated diagnostic approaches combining clinical, demographic, and laboratory data to improve early detection in resource-limited settings.

**What this study adds:**

Although cost-effective and widely available, gamma gap and AGR have limited accuracy for screening monoclonal gammopathies when used alone in settings with prevalent infectious and inflammatory diseases. Although the tests are good at ruling out monoclonality, they risk missing many true cases, delaying diagnosis and treatment.

## Introduction

Monoclonal gammopathies include both malignant conditions, such as multiple myeloma, plasmacytoma, and Waldenström macroglobulinaemia, as well as the pre-malignant condition, monoclonal gammopathy of undetermined significance (MGUS).^1^ These disorders are characterised by the abnormal proliferation of clonal plasma cells producing monoclonal immunoglobulins. Multiple myeloma, the most serious and well-known form, often progresses from MGUS and leads to complications such as anaemia, renal dysfunction, osteolytic lesions, and increased susceptibility to infections. Despite advances in treatment, multiple myeloma remains incurable but manageable.^2^

Multiple myeloma accounts for 1.3% of all malignancies and 15% of haematological malignancies, with an estimated 138 000 new cases diagnosed globally each year.^3^ However, the incidence and mortality rates are significantly higher in affluent regions like North America, Western Europe, and Australia compared to less affluent regions such as sub-Saharan Africa, Asia, and Oceania.^3^ Cowan et al.^3^ and Rebbeck et al.^4^ suggest that this disparity may result from limited access to screening and diagnostic tools in resource-constrained settings. Consequently, many cases of multiple myeloma go undetected or are diagnosed at advanced stages, leading to an underestimation of the true prevalence in regions such as South Africa.^3,4,5^

Screening for monoclonal gammopathies is critical for early detection, enabling timely intervention and reducing the risk of complications.^6,7^ This is particularly important in low-resource settings, where restricted access to advanced diagnostic tools contributes to delayed diagnoses and poorer outcomes.^3,4^ Current screening and diagnostic methods, such as serum protein electrophoresis (SPE), immunofixation, serum-free light chains (SFLC), urine protein electrophoresis, and bone marrow biopsy, while effective, are costly and complex,^8^ limiting their accessibility in resource-poor environments.^1^ This underscores the need for simple, affordable, and reliable screening tools to determine the need for further investigation.

The gamma gap and albumin-to-globulin ratio (AGR), derived from routinely measured total protein and albumin concentrations, have been used as clinical screening tools for monoclonal gammopathies.^9,10,11^ Their low cost and ease of calculation make them appealing options. However, their diagnostic performance, particularly across diverse populations, remains inadequately established. Additionally, chronic infections, inflammatory conditions, and malnutrition can influence the gamma gap and AGR, leading to inaccurate results.^12,13,14^

In regions such as sub-Saharan Africa, where the burden of infectious and inflammatory diseases is high, the gamma gap and AGR may perform poorly as screening tools. South Africa faces a significant burden of infectious and inflammatory diseases, including high rates of HIV, tuberculosis, viral hepatitis, and other chronic infections.^15,16^ This can result in missed true cases, delayed diagnoses, or wasted healthcare resources resulting from unnecessary investigations and follow-up for false positives, limiting the tools’ effectiveness. Understanding these limitations is crucial to avoiding misdiagnoses and to optimising healthcare resources in already overburdened systems.

Assessing the performance of screening and diagnostic tools is crucial to guiding their clinical use and to support the development of more reliable methods. This study aimed to evaluate the diagnostic accuracy of the gamma gap and AGR in identifying patients who required further investigation for monoclonal gammopathies in a population burdened by inflammatory and infectious diseases. We hypothesised that the two metrics would perform poorly within this population.

## Methods

### Ethical considerations

Ethical approval was obtained from the Human Research Ethics Committee of the University of Cape Town (reference no. HREC: 589/2023). Data for this study were managed in accordance with the data management plan, adhering to data protection and privacy regulations. Only data required for analysis were extracted and securely stored, and access was limited to authors. Informed consent for individual inclusion was waived because of the use of data for secondary research purposes.^17^ The study adhered to international ethical standards, including the Declaration of Helsinki.

### Study design

This retrospective, observational study evaluated the diagnostic accuracy of the gamma gap and AGR in classifying patients with suspected monoclonal gammopathies, with the goal of determining their effectiveness as screening tools in a real-world clinical setting.

### Setting

Groote Schuur Hospital is an 893-bed tertiary teaching hospital in Cape Town, South Africa. The hospital’s National Health Laboratory Service laboratory is accredited to the ISO 15189 standard, affiliated with the University of Cape Town, and provides extensive testing for monoclonal gammopathies for the public sector.

### Data collection

The data used in this study were extracted from the Monoclonal Gammopathy Research Group – University of Cape Town Database (HREC: R042/2022) for subsequent analysis.

### Study population

The database comprised de-identified retrospective data for adult patients (≥ 18 years) who underwent investigations for monoclonal gammopathies in our laboratory. The data, sourced from the laboratory information system and analysers, included basic demographics (age and sex) and results from SPE, immunofixation, total protein, and albumin conducted from 01 September 2015 to 30 September 2022. All patients with complete biochemical data were considered for inclusion.

Exclusion criteria included missing data or equivocal immunofixation results, necessary to classify patients into two outcome groups. Cancelled tests, duplicate tests, and follow-up visits were also excluded to prevent verification or spectrum bias. Hypogammaglobulinaemic patients underwent routine immunofixation to rule out small but clinically significant monoclonal proteins or interference. These samples were included in primary analysis but were excluded in sub-group analysis for comparison.

### Laboratory analyses

Data were generated using routine clinical laboratory analysers and methods. The SPE was performed using capillary zone electrophoresis on the Minicap, and immunofixation was performed using agarose gel electrophoresis on the Hydrasys 2 (both Sebia, Lisses, France). Albumin and total protein were measured on the Roche cobas© 6000 analyser (Roche Diagnostics GmbH, Mannheim, Germany) with a coefficient of variation of 5.02% for albumin and 3.95% for total protein. The laboratory processes SPE requests from clinicians, and immunofixation is reflexively added by the pathologist based on clinical and biochemical information, detection of suspicious peaks, or the presence of hypogammaglobulinaemia.

### Data analysis

The Monoclonal Gammopathy Research Group – University of Cape Town Database underwent a curation pipeline for checking and cleaning, with quality control checks to ensure accuracy and reliability for this study. Statistical analyses were performed using Python-based packages (Python Software Foundation, Beaverton, Oregon, United States) in PyCharm (JetBrains, Prague, Czech Republic).

**FIGURE 1:**
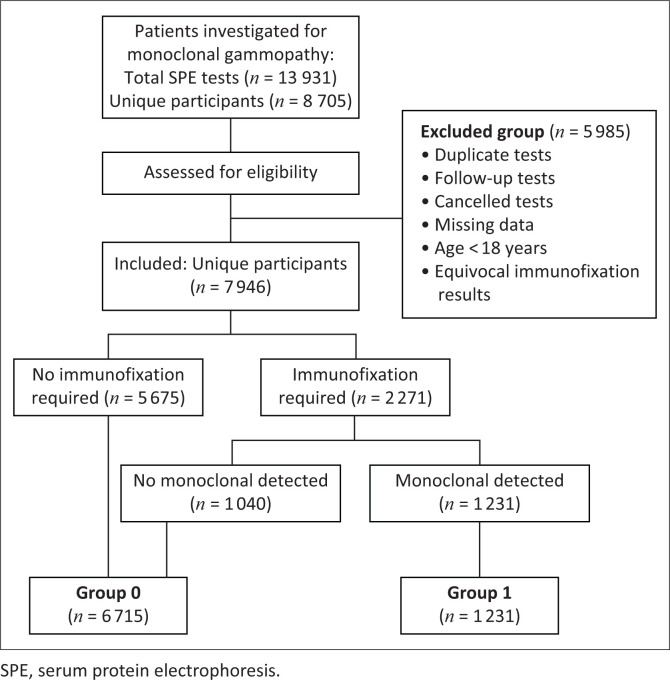
Flowchart depicting the stepwise categorisation of patients evaluated for monoclonal gammopathy at a tertiary hospital laboratory in South Africa from 01 September 2015 to 30 September 2022. The patients were categorised into ‘Group 0’ (no monoclonal protein detected), ‘Group 1’ (monoclonal protein detected), and the ‘Excluded Group’ (exclusion criterion met). A total of 13 931 SPEs were assessed for eligibility. After excluding 5985 tests, 7946 unique patients were included. Patients were then classified by immunofixation. 5675 patients did not require immunofixation and were assigned to Group 0. From immunofixation, 1040 were negative for monoclonality and thus also categorised into Group 0. Positive immunofixation results categorised patients into Group 1.

Figure 1 shows the flow of patients, and the algorithm used to categorise patients into two groups (‘Group 0’ [no monoclonal protein] and ‘Group 1’ [monoclonal protein detected]). Patients were initially classified into three categories based on their SPE results and local policy. Those with ‘unremarkable’ SPEs, showing no suspicious peaks or irregularities (< 1 g/L), were placed in Group 0. Patients whose SPE showed a suspicious peak required immunofixation and, based on the results, were placed into either Group 0 or Group 1. Patients with equivocal immunofixation results were excluded to prevent misclassification.

The gamma gap was calculated as the difference between total protein and albumin concentrations (g/L). The AGR was calculated as albumin divided by globulin, where globulin was equivalent to the gamma gap.

Normality of continuous variables was assessed using histograms, quantile-quantile plots, and the Anderson-Darling test. Categorical variables were described as numbers, proportions, and percentages, and visualised using pie charts (Online Supplementary Figure 1). Continuous variables were expressed as median (interquartile range), and illustrated with histograms (Online Supplementary Figure 2). Chi-squared tests were used for categorical variables, and Wilcoxon rank-sum for continuous variables. A *p*-value < 0.05 was considered significant.

**FIGURE 2:**
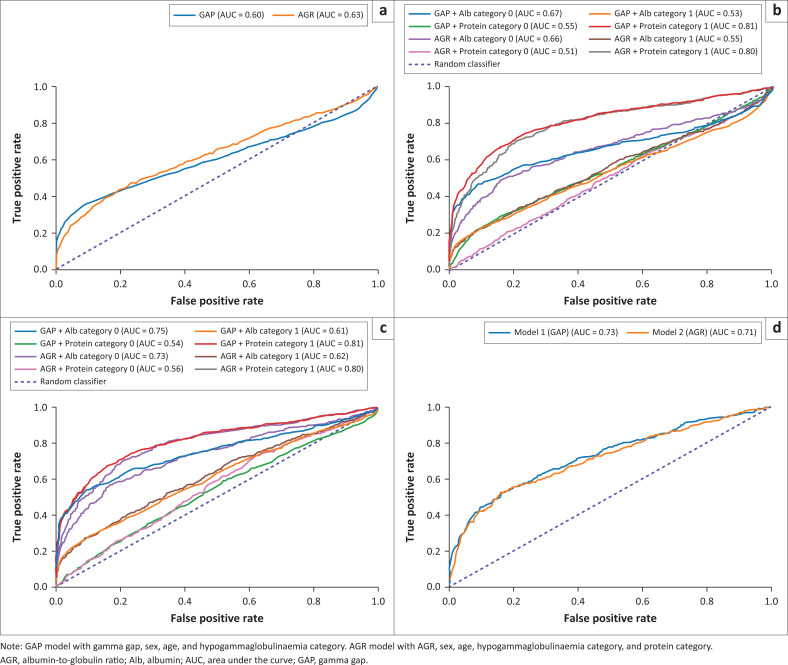
Receiver operating characteristic curves illustrating the ability of the gamma gap and albumin-to-globulin ratio (AGR) to predict monoclonality in patients being evaluated for monoclonal gammopathy at a tertiary hospital laboratory in South Africa from 01 September 2015 to 30 September 2022. (a) In the whole population; (b) by albumin (< 35 g/L) and protein (> 78 g/L) categories in the whole population; (c) by albumin and protein categories excluding hypogammaglobulinaemic patients; (d) illustrates the performance of the multivariable logistic regression models.

Continuous variables were *z*-score normalised, and categorical variables were encoded. Receiver operating characteristic (ROC) curves were constructed to evaluate diagnostic performance of gamma gap, AGR, and logistic regression models, with area under the curve (AUC) used as a measure of accuracy (Online Supplementary Text 1). The optimal cut-offs were determined using the Youden Index, and sensitivity and specificity were calculated at these cut-offs.

Univariable logistic regression was performed to identify predictors of the outcome. Multivariable models were then developed and optimised. Akaike Information Criterion (AIC) values were calculated to compare univariable and multivariable models. Confusion matrices and classification reports were generated to assess model performance.

Subgroup analyses were performed to further evaluate factors that may influence metric performance. Patients with hypogammaglobulinaemia were excluded, and subgroups based on albumin (< 35 g/L vs ≥ 35 g/L) and total protein (≤ 78 g/L vs > 78 g/L) categories, based on their respective reference intervals, were also examined.

## Results

### Patient characteristics

A total of 13 931 records from 8705 unique patients were evaluated for eligibility (Figure 1). After applying the inclusion and exclusion criteria, 5985 records were removed, leaving 7946 unique patients. These patients were categorised into ‘Group 0’ (no monoclonal detected, *n* = 6715) and ‘Group 1’ (monoclonal detected, *n* = 1231).

Continuous variables were distributed non-parametrically. A total of 58% of patients were female and 42% were male patients (Table 1). Sex distribution was more balanced (54% women and 46% men) among patients with monoclonality. The median (p25, p75) age was 61 (50, 71) years.

**TABLE 1:** Population characteristics of patients evaluated for monoclonal gammopathy at a tertiary hospital laboratory in South Africa from 01 September 2015 to 30 September 2022.

Variables	Total (*N* = 7946)				Group 0 (*n* = 6713)				Group 1 (*n* = 1233)				*p*	
	*n*	%	Median	p25, p75	*n*	%	Median	p25, p75	*n*	%	Median	p25, p75	Mann-Whitney *U*	*χ* ^2^
**Age (years)**	-	-	61	50, 71	60	-	60	49, 70	-	-	63	52, 72	< 0.001*	-
**Sex**	-	-	-	-	-	-	-	-	-	-	-	-	-	< 0.001*
Female patients	4632	58	-	-	3970	59	-	-	662	54	-	-	-	-
Male patients	3314	42	-	-	2743	41	-	-	571	46	-	-	-	-
**Total protein (g/L)**	-	-	73	67, 79	-	-	73	67, 78	-	-	75	66, 89	< 0.001*	-
**Albumin (g/L)**	-	-	40	33, 44	-	-	40	34, 44	-	-	36	28, 41	< 0.001*	-
**Gamma gap (g/L) †**	-	-	33	28, 39	-	-	32	28, 38	-	-	36	28, 55	< 0.001*	-
**AGR ‡**	-	-	1.20	0.87, 1.50	-	-	1.23	0.92, 1.52	-	-	0.97	0.54, 1.39	< 0.001*	-
**Hypogammaglobulinaemia**	615	-	-	-	364	59	-	-	251	41	-	-	-	< 0.001*
**Albumin < 35 (g/L)**	2450	31	-	-	1881	28	-	-	569	46	-	-	-	< 0.001*
**Total protein > 78 (g/L)**	2114	27	-	-	1599	24	-	-	515	42	-	-	-	< 0.001*

Note: Group 0: No monoclonal detected; Group 1: Monoclonal detected.

AGR, albumin-to-globulin ratio; p25, 25th percentile; p75, 75th percentile.

* , *p* < 0.05 was deemed significant.

† , Gamma Gap = Total Protein – Albumin;

‡ , AGR, Albumin-to-Globulin Ratio = Albumin / Globulin, where Globulin = Total Protein – Albumin.

Total protein was higher in the monoclonal compared to no monoclonal detected group (75 [66, 89] vs 73 [67, 78] g/L, *p* < 0.001), while albumin was lower (36 [28, 41] vs 40 [34, 44] g/L, *p* < 0.001). The gamma gap was higher in the monoclonal group (36 [28, 55] vs 32 [28, 38], *p* < 0.001), while the AGR was lower (0.97 [0.54, 1.39] vs 1.23 [0.92, 1.52], *p* < 0.001).

Among 615 hypogammaglobulinaemic patients, 41% had a monoclonal protein, while 59% did not (*p* < 0.001) (Online Supplementary Table 1). Approximately one-third of patients had low albumin (< 35 g/L; reference interval: 35 g/L – 52 g/L) and one-third had high total protein (> 78 g/L; reference interval: 60 g/L – 78 g/L).

### Receiver operating characteristic curves

The gamma gap demonstrated an AUC of 0.60 (95% CI: 0.58–0.62), while AGR (0.63 [95% CI: 0.61–0.65]) was slightly higher (Table 2; Figure 2a). At an optimal cutoff of 46 g/L for the gamma gap, sensitivity was 35% (95% CI: 33–38) and specificity was 91% (95% CI: 90–92). For AGR, an optimal cutoff of 0.85 had a sensitivity of 44% (95% CI: 41–47) and specificity of 80% (95% CI: 79–81).

**TABLE 2:** Diagnostic accuracy of gamma gap, albumin-to-globulin ratio, and multivariable logistic regression models for predicting monoclonality in patients evaluated for monoclonal gammopathy at a tertiary hospital laboratory in South Africa from 01 September 2015 to 30 September 2022.

Description	ROC		Optimal cutoff	Sensitivity		Specificity	
	AUC	95% CI		%	95% CI	%	95% CI
**GAP**							
**Hypogamma included**							
Whole population	0.60	0.59–0.62	46	35	33–38	91	90–92
Albumin < 35	0.67	0.65–0.69	55	47	43–51	91	90–92
Albumin ≥ 35	0.54	0.50–0.56	41	24	21–27	90	89–91
Protein ≤ 78	0.55	0.53–0.58	45	30	26–33	84	83–85
Protein > 78	0.81	0.78–0.84	51	68	64–72	84	82–86
**Hypogamma excluded**							
Sub-population	0.68	0.66–0.70	46	42	38–45	91	90–91
Albumin < 35	0.75	0.72–0.77	55	54	50–59	90	89–92
Albumin ≥ 35	0.61	0.58–0.64	41	29	25–33	89	88–90
Protein ≤ 78	0.54	0.50–0.57	31	59	55–64	48	47–49
Protein > 78	0.81	0.78–0.84	51	67	63–72	84	82–86
**GAP LogReg model†**							
Whole population	0.73	0.70–0.76	0.154	58	53–63	78	76–80
**AGR**							
**Hypogamma included**							
Whole population	0.63	0.61–0.65	0.85	44	41–47	80	79–81
Albumin < 35	0.66	0.64–0.69	0.51	49	45–53	84	82–86
Albumin ≥ 35	0.55	0.53–0.58	0.95	22	19–26	91	90–92
Protein ≤ 78	0.51	0.48–0.53	0.85	21	18–24	82	81–83
Protein > 78	0.80	0.77–0.82	0.74	71	67–74	79	77–81
**Hypogamma excluded**							
Sub-population	0.68	0.66–0.70	0.85	50	47–54	79	78–80
Albumin < 35	0.73	0.71–0.76	0.51	57	52–61	83	81–85
Albumin ≥ 35	0.62	0.59–0.65	1.09	39	35–43	79	78–80
Protein ≤ 78	0.56	0.53–0.58	1.40	73	68–76	39	37–40
Protein > 78	0.80	0.77–0.82	0.74	70	66–74	79	77–81
**AGR LogReg model** ‡							
Whole population	0.71	0.68–0.74	0.203	55	50–60	81	79–83

Note: The GAP and AGR model calculations can be found in Online Supplementary Text 1 of Khumalo N, Francis CA, Baloyi SM, Rusch JA. Gamma gap and albumin-globulin ratio show poor sensitivity for monoclonal gammopathy screening in South Africa. Afr J Lab Med. 2025;14(1), a2505. https://doi.org/10.4102/ajlm.v14i1.2505

AGR, albumin-to-globulin ratio; AUC, area under the curve; GAP, gamma gap; Hypogamma, hypogammaglobulinaemia; LogReg, logistic regression; ROC, receiver operating characteristic.

† , logistic regression model including gamma gap, age, sex, albumin category (< 35 g/L or ≥ 35 g/L), total protein category (> 78 or ≤ 78), and hypogamma (binary);

‡ , logistic regression model including AGR, age, sex, albumin category (< 35 g/L or ≥ 35 g/L), total protein category (> 78 or ≤ 78), and hypogamma (binary).

Including hypogammaglobulinaemic patients detracted from the predictive power of both metrics. Excluding these patients improved the AUC to 0.68 for both. Performance was further influenced by clinically relevant albumin and total protein categories (Table 2; Figure 2b and 2c). The gamma gap and AGR performed better in patients with low albumin (AUC both 0.67) and even better when hypogammaglobulinaemic patients were excluded (AUC 0.75 and 0.73). Both metrics showed improved performance in patients with high total protein (AUC 0.81 and 0.80). Excluding hypogammaglobulinaemic patients did not affect performance in this subgroup. Conversely, the metrics performed more poorly in subgroups with normal/raised albumin and low/normal total protein. Excluding hypogammaglobulinaemic patients from these analyses improved the performance in the normal/raised albumin group (AUC 0.61 and 0.62).

### Logistic regression models

Two multivariable models were developed for the gamma gap and AGR using an iterative process. Univariable logistic regression indicated that gamma gap, AGR, age, albumin, and total protein, as well as the categories sex, albumin, protein, and hypogammaglobulinaemia, were all predictors of the outcome (all *p* < 0.001; Online Supplementary Table 2).

During development, high correlation between gamma gap, AGR, total protein, and albumin prompted development of two separate multivariable models: the ‘GAP’ model and the AGR model. Initially, the GAP model included gamma gap, age, sex, and categories for albumin, total protein, and hypogammaglobulinaemia. During optimisation, albumin and protein categories were excluded – adding complexity without improving predictive power (Online Supplementary Table 3). Similarly, the albumin category was excluded from the AGR model.

The final GAP model, based on 5578 observations, converged successfully and explained 18% of the variance (Pseudo *R*^2^ = 0.180), while log-likelihood was −1984.5, outperforming the null model (−2419.9). The likelihood ratio test confirmed the joint significance of predictors (*p* < 0.0001). The GAP model achieved AUC 0.73, sensitivity 58% and specificity 78% (Table 2; Figure 2d). The AGR model performed similarly, with AUC 0.71 (sensitivity 55% and specificity 81%).

We then assessed how well the gamma gap and AGR could predict hypogammaglobulinaemia (Online Supplementary Table 4). At an optimal cutoff of 27 g/L, the gamma gap performed well, with AUC 0.86, sensitivity 80% and specificity 83%. The AGR was considerably less sensitive (57%) and specific (75%), with AUC 0.71.

### Model comparison

The performance of the two multivariable models was compared with the univariable models for gamma gap and AGR. The AIC values were 3979 for the multivariable GAP model and 4211 for the AGR model, suggesting that the GAP model provided a better balance between goodness-of-fit and complexity. In contrast, the AIC values for the univariable models were much higher (6320 for the GAP model, and 6655 for the AGR model), indicating that the multivariable models offered superior performance despite their complexity.

Confusion matrices and classification reports showed that multivariable models achieved higher accuracy, with GAP and AGR models achieving 75% and 78% accuracy, compared to 69% and 75% for the univariable models, respectively (Online Supplementary Figure 3; Online Supplementary Table 5).

## Discussion

Our findings highlight that the gamma gap and AGR provide limited diagnostic accuracy for identifying monoclonal gammopathies in real-world clinical settings with a high burden of inflammatory and infectious diseases, such as in South Africa. The low sensitivity of these tests indicates a significant number of positive cases may go undetected, delaying diagnosis and treatment. However, their high specificity ensures accurate identification of most patients without the condition, reducing false positives and unnecessary further investigations. While this enhances their utility for ruling out cases, sensitivity should be prioritised for screening purposes to ensure all cases are captured.

The sub-group analyses we performed offer further insights. The diagnostic performance of the gamma gap and AGR improved when albumin and total protein concentration categories were considered. Including hypogammaglobulinaemic patients in the analysis confounded the results, as 41% of these patients had monoclonal proteins despite significantly lower total protein and gamma gap values. Notably, a low gamma gap (< 27 g/L) was predictive of hypogammaglobulinaemia, indicating that both high and low gamma gap values should raise suspicion for monoclonality in appropriate clinical contexts.

Previous studies have shown disparities in diagnostic performance across different contexts. In an Australian study, Thakkinstian et al. reported higher accuracy for the gamma gap, with an odds ratio of 4.5 (95% CI: 3.8–5.4) at a threshold of 41 g/L.^10^ Similarly, in a study performed at Duke University Medical Center in the United States, Dupuis et al. demonstrated a strong correlation between the gamma gap and monoclonal protein concentrations, emphasising its potential as a rapid, simple test in resource-limited settings.^11^ Unlike this present study, both studies were conducted in well-resourced settings and did not address the influence of inflammatory and infectious diseases on test performance. In contrast, Liu et al.^13^ observed limited sensitivity but high specificity for the gamma gap (AUC of 0.64 [95% CI: 0.60–0.69], sensitivity of 15.4%, specificity of 95.4%, threshold of 40 g/L) in a United States community-based study that included patients with MGUS, HIV, and hepatitis C. These findings align with our results, suggesting that diagnostic performance is compromised in settings with a high burden of infectious and inflammatory conditions.

While we found that the gamma gap and AGR perform similarly, few studies have specifically evaluated the AGR in this context. A study by Tarín-Arzaga et al.^18^ in Mexico included all SPE results (*n* = 1578) from 255 individuals and found that the AGR (AUC = 0.74, sensitivity = 59%, specificity = 79%) performed better than the gamma gap (AUC = 0.71, sensitivity = 52%, specificity = 80%) overall.^18^ However, when predicting a monoclonal protein ≥ 10 g/L, both metrics showed excellent diagnostic performance, with an AUC of 0.94, and comparable sensitivity (84% vs 87%) and specificity (87% vs 84%) values, respectively.

In South Africa, where infectious and inflammatory diseases are prevalent,^15,16^ conditions such as HIV, tuberculosis, chronic hepatitis (B and C), rheumatoid arthritis, and systemic lupus erythematosus compete for attention in the differential diagnosis of monoclonal gammopathy. These conditions are often associated with abnormal protein levels and polyclonal hypergammaglobulinaemia. These changes increase the gamma gap and lower the AGR, complicating clinical decisions on diagnostic pathways and additional testing, in resource-limited settings. Careful clinical evaluation is essential to differentiate monoclonal gammopathy from these prevalent conditions.

Studies have reported inconsistent gamma gap thresholds. Similar to our findings, a Canadian study by Suleman et al. found a low sensitivity (47.7%) but moderate specificity (77.1%) at a threshold of 40 g/L.^19^ When they evaluated the threshold of 44 g/L, the specificity improved to 89.2%, at the expense of sensitivity (33.7%).^19^ In contrast, Pawar and Hedge in India observed high sensitivity (82.1%) and specificity (85.4%) at a lower threshold of 32.5 g/L, concluding that the gamma gap reliably distinguishes monoclonality.^20^ However, their findings are limited by a small sample size (*n* = 147), despite being conducted in a similar low-to middle-income country setting with a high burden of infectious and inflammatory diseases (*n* = 147). Furthermore, Hassan and Hameed in Iraq found that, at a threshold of 29.1 g/L, the gamma gap distinguished treated from untreated multiple myeloma patients with an AUC of 0.735 (95% CI: 0.66–0.904, *p* < 0.001), achieving 76.5% sensitivity and 70.6% specificity.^21^

In our study, multivariable models outperformed univariable models, although both demonstrated low precision and recall in detecting monoclonal cases, with the AGR model performing slightly better. The GAP model, however, showed a significantly lower AIC value, indicating a better balance between model fit and simplicity. Despite this, both models have limited diagnostic utility in isolation and would benefit from further optimisation by incorporating additional predictive factors. This highlights the importance of a comprehensive approach to accurately predict monoclonal gammopathies,^13^ as demonstrated by Thakkinstian et al., who used five variables (gamma gap, age, sex, haemoglobin, and eGFR) in their optimised model, achieving an AUC of 0.80.^10^ This study was strengthened by confirmation of their findings in a validation data set (*n* = 4312).

### Recommendations

Universal screening for monoclonal gammopathy in asymptomatic individuals is not recommended.^20^ This study aimed to evaluate the gamma gap and AGR as supplementary tools to identify patients at risk of monoclonal gammopathy, rather than replacing specialised tests such as SPE, immunofixation, SFLC, or urine protein electrophoresis. These commonly requested and cost-effective tests may aid in risk stratification. Evidence has consistently shown that combining tests enhances diagnostic sensitivity in monoclonal detection, with a combination of SPE, immunofixation, and SFLC providing the most accurate diagnostic outcomes.^21,22,23,24,25^

### Strengths

This study leveraged a large real-world sample from a high-burden setting, with tests conducted on standard equipment and reviewed by pathologists, ensuring high-quality data.

### Limitations

Several limitations may affect the generalisability of the findings, including the focus on patients already under investigation for monoclonal gammopathy, retrospective design, and absence of clinical details like comorbidities or medication use. Excluding patients with equivocal immunofixation results, while reducing misclassification, further limits applicability in cases of inflammation or unclear findings.

### Conclusion

Neither the gamma gap nor the AGR demonstrates sufficient reliability as standalone screening tools for monoclonal gammopathy in settings with a high burden of infectious and inflammatory diseases. While both metrics showed moderate specificity, their low sensitivity highlights the risk of missed cases. These findings emphasise the need for a more integrated approach that combines clinical, demographic, and laboratory data, particularly in resource-limited settings. Further research is essential to develop more effective screening strategies and explore the potential of combining these markers with other clinical parameters to improve diagnostic performance.
